# Effect of stimulator of interferon genes (STING) signaling on radiation-induced chemokine expression in human osteosarcoma cells

**DOI:** 10.1371/journal.pone.0284645

**Published:** 2023-04-20

**Authors:** Sita S. Withers, Cambri E. Moeller, Cally N. Quick, Chin-Chi Liu, Shelby M. Baham, Jayme S. Looper, Ramesh Subramanian, Konstantin G. Kousoulas

**Affiliations:** 1 Department of Veterinary Clinical Sciences, School of Veterinary Medicine, Louisiana State University, Baton Rouge, Louisiana, United States of America; 2 Department of Pathobiological Sciences, School of Veterinary Medicine, Louisiana State University, Baton Rouge, Louisiana, United States of America; University of Cincinnati, UNITED STATES

## Abstract

Cancer cell-intrinsic mechanisms affecting radiation immunomodulation could be exploited to optimize systemic effects of localized radiation. Radiation-induced DNA damage is sensed by cyclic GMP-AMP synthase (cGAS), which ultimately activates stimulator of interferon (IFN) genes (STING). Resultant expression of soluble mediators such as CCL5 and CXCL10 can facilitate recruitment of dendritic cells and immune effector cells into the tumor. The primary objectives of this study were to determine the baseline expression levels of cGAS and STING in OSA cells and evaluate the dependence of OSA cells on STING signaling for eliciting radiation-induced expression of CCL5 and CXCL10. cGAS and STING expression, and CCL5/CXCL10 expression in control cells, STING-agonist treated cells, and cells treated with 5 Gy ionizing radiation were assessed utilizing RTqPCR, Western blot, and ELISA. U2OS and SAOS-2 OSA cells were deficient in STING relative to human osteoblasts (hObs), while SAOS-2-LM6 and MG63 OSA cells expressed equivalent amounts of STING compared to hObs. A dependence on baseline or induced STING expression was observed for STING-agonist, and radiation-induced, expression of CCL5 and CXCL10. This finding was confirmed by performing siRNA knockdown of STING in MG63 cells. These results show that STING signaling is necessary for radiation-induced expression of CCL5 and CXCL10 in OSA cells. Additional studies are necessary to determine whether STING expression in OSA cells *in vivo* alters immune cell infiltrates after radiation exposure. These data may also have implications for other potentially STING-dependent characteristics such as resistance to oncolytic virus cytotoxicity.

## Introduction

The lack of significant advancements in osteosarcoma (OSA) treatment over the last 30 years has generated interest in harnessing anticancer immunity to attempt to address the high rate of OSA metastasis [1, 2]. Induction of an anticancer immune response is typically dependent on tumor infiltration by activated, tumor-targeting immune-effector cells such as CD8+ T lymphocytes (T cells) and natural killer (NK) cells [3]. Several studies have shown that activation and infiltration of CD8+ cytotoxic T cells and NK cells are reliant on type I interferon (IFN) such as IFNβ, and chemokines such as CCL5 and CXCL10 in the tumor microenvironment [4–9].

One of the major pathways regulating type I IFN production is stimulator of IFN genes (STING) [6, 10]. After the detection of cytoplasmic DNA, cyclic GMP-AMP (cGAMP) synthase (cGAS) catalyzes the formation of cGAMP, which then interacts with STING to recruit transcription factors such as IFN regulatory factor 3 (IRF3) and others. What follows is the upregulation of type I IFN such as IFNβ and additional innate and adaptive signals such as CCL5, CXCL10 and others [6, 11]. Consequentially, inherent genomic instability or radiation exposure can result in the detection of cytoplasmic DNA by cGAS, and this pathway is necessary for radiation-induced systemic anti-cancer immunity [4, 12–14]. This phenomenon, termed the abscopal effect, can mediate therapeutic responses in cancer tissues distant to the radiation field.

A previous report revealing that two human OSA cell lines expressed very low levels of STING raises the question of whether STING deficiency in OSA could mediate immune resistance of this tumor type [15]. Therefore, the objectives of this study were to 1) evaluate the variability of STING expression in human OSA cell lines relative to normal human osteoblasts (hObs), and 2) to determine the reliance of OSA cells on STING expression for inducing innate inflammatory signals and chemokine expression after radiation exposure. We report that STING signaling is necessary for radiation-induced expression of CCL5 and CXCL10 in OSA cells. These data further our understanding of factors affecting radiation-induced immunomodulation in OSA cells.

## Materials and methods

### Cell culture and reagents

Human osteoblasts (hObs) were purchased from, and authenticated by, Cell Applications (#406–05, San Diego, CA, USA), and human OSA cell lines (SAOS-2 (SAOS), SAOS-2-LM6 (LM6), MG63, U2OS) were purchased from the MD Anderson Characterized Cell Line Core (Houston, TX). hObs were cultured in human osteoblast growth medium (#417–500, Cell Applications, San Diego, CA, USA), and OSA cell lines were cultured in complete media consisting of Dulbecco’s Modified Eagles Medium (DMEM, SH30022.01, Cytiva, Logan, UT, USA) supplemented with 10% Fetal Bovine Serum (FBS, 83007–198, VWR) and 1% penicillin/streptomycin (K952-100ML, VWR, Sanborn, NY, USA). Cells were incubated at 37°C, with 5% CO_2_. Mammalian (non-canonical) CDN, cyclic[G(2’,5’)pA(3’,5’)p] (2’3’-cGAMP, #tlrl-nacga23-02, InvivoGen, San Diego, CA, USA) was reconstituted using 1.5 mL endotoxin-free water.

For baseline mRNA and protein expression, cells were processed when they achieved ~90% confluency in a 6-well plate. For cGAMP stimulation experiments, cells were plated in 6-well plates at counts of 7 x 10^5^ cells/well for SAOS, and 5 x 10^5^ cells/well for LM6, MG63, and U2OS. After resting overnight, cells were then treated with 10 μg/ml cGAMP or control media. RNA was extracted after 16 hrs, and cell culture supernatant was collected after 24 hrs.

### Total RNA extraction

The RNeasy mini kit (74106, Qiagen, Germantown, MD, USA) was utilized for recovery of total RNA. Adherent cells were washed with Dulbecco’s phosphate buffered saline (DPBS, 10010–031, Gibco, Carlsbad, CA, USA) and briefly incubated with 0.25% Trypsin-EDTA (trypsin, 25200–056, Gibco, Grand Island, NY, USA). Detached cells were then washed and centrifuged for 5 min at 400 g and 4°C. 350 μL of lysis buffer (Buffer RLT, 10155762, Qiagen) was added to the pelleted cells, which was then vortexed for 1 min to homogenize the sample. The remainder of the RNA extraction was performed per protocol (RNeasy mini kit, Qiagen). RNA was quantified and checked for purity using the NanoDrop One Spectrophotometer (Thermo Scientific) and immediately stored at -80°C. Only samples with adequate purity (A260/A230 and A260/A280 > 1.8) were used for downstream applications.

### Reverse transcription-quantitative PCR (rt-qPCR)

RNA was converted into cDNA using the qScript cDNA Synthesis Kit (95047–100, Quanta BioSciences, Gaithersburg, MD, USA) according to the manufacturer’s protocol. Total RNA (1 μg), qScript reagent (4 μL) and reverse transcriptase (1 μL) were combined into 0.2 ml sterile PCR reaction tubes. The cDNA reaction was performed using the GeneAmp PCR System 9700 thermal cycler (PE Applied Biosystems) using the parameters: 5 minutes at 25°C, 30 minutes at 42°C, 5 minutes at 85°C, and hold at 4°C. RT-qPCR reactions were prepared according to the manufacturer’s protocol using PerfeCTa SYBR Green FastMix ROX (95073–012, Quanta BioSciences, Gaithersburg, MD, USA). Primer pairs targeting TBP, STING, cGAS, CCL5, and CXCL10 were synthesized by IDT (Integrated DNA Technologies, San Diego, CA, USA), and details are provided in S1 Table. qPCR reactions were performed in duplicate in a 384 well plate with a reaction volume of 10 μL per well (2 μL cDNA, 0.4 μL forward and reverse primers, 5 μL PerfeCTa SYBR Green FastMix, 2.2 μL nuclease-free water).

The reactions were run on a 7900HT Fast Real-Time PCR System (Applied Biosystems), using the recommended cycle conditions for PerfeCTa SYBR Green FastMix ROX kit: hold at 95°C for 60s, then 40 cycles of 95°C for 15s to 60°C for 60s, and finishing with a dissociation curve. Reactions were performed in duplicate and a “no template control” reaction was performed for all primer pairs to ensure no DNA contamination of reagents. Dissociation curves were reviewed for each reaction to confirm amplification of a single product. TBP was selected as the reference gene based on minimal regulation under various study conditions, and was used for ΔCT calculations. Relative expression of treated samples were compared to control samples via the ΔΔCT method. Fold change was expressed as 2^-ΔΔCT^. Log_2_(fold change) was used for statistical analysis. Findings were repeated at least twice, from cell culture through RT-qPCR, to ensure reproducibility.

### Enzyme-linked immunosorbent assay (ELISA)

Supernatant from OSA cells incubated with 10 μg/ml 2’3’-cGAMP or control media for 24 hours was collected and centrifuged at 2000 g for 10 minutes at 4°C, aliquoted, and frozen at -80°C. The concentrations of CXCL10 and CCL5, were measured using human CXCL10/IP-10 ELISA kit (Quantikine, DIP100, R&D Systems, Minneapolis, MN, USA) and human CCL5/RANTES ELISA kits (Quantikine, DRN00B, R&D Systems, Minneapolis, MN, USA), respectively. Assays were performed in duplicate per manufacturer protocols, and 2–3 independent replicates were performed.

### Western blot

Adherent cells were washed with ice cold DPBS twice, and then incubated with Pierce RIPA buffer (89900, ThermoFisher Scientific, Waltham, MA, USA) containing 1x protease/phosphatase inhibitor (Halt Protease and Phosphatase Inhibitor Cocktail; 78440, ThermoFisher Scientific, Waltham, MA, USA) for 3 mins on ice. A cell scraper was used to collect the cell lysate and samples were stored at -80°C. Protein concentration was determined using Pierce BCA protein assay kit (23225, ThermoFisher Scientific, Waltham, MA, USA) per the manufacturer’s protocol. 40 μg protein was then combined with 1x Laemmli sample buffer (1610747, Bio-Rad, Hercules, CA, USA), incubated at 95°C for 5 mins, and loaded onto a 10% Mini-PROTEAN TGX precast polyacrylamide gel (4561034, Bio-Rad, Hercules, CA, USA). PageRuler Plus Prestained Protein Ladder (26620, ThermoFisher Scientific, Waltham, MA, USA) was added to one well. 1x Tris/Glycine/SDS running buffer (1610732, Bio-Rad, Hercules, CA, USA) was used for electrophoresis under the following conditions: 50 V for 15 mins, 90 V for 20 mins, and 150 V until complete. 1x Tris/Glycine/SDS transfer buffer (containing 20mM Tris Base, 0.1% SDS, 1.13% glycine, 20% ethanol) was used to transfer to a polyvinylidene difluoride (PVDF) membrane (162–0177, Bio-Rad, Hercules, CA, USA) at 300mA for 90 mins on ice. Membranes were incubated in anti-human STING monoclonal antibody (~33 & 35 kDa, clone D2P2F, #13647S, Cell Signaling, Danvers, MA, USA) at a ratio of 1:500, or anti-human cGAS monoclonal antibody (~62 kDa, clone D1D3G, #15102S, Cell Signaling, Danvers, MA, USA) at a ratio of 1:500, with 2% non-fat milk overnight at 4°C. They were then washed 3 times using 1x PBST and incubated in IgG (H+L) Cross-Adsorbed Mouse anti-Rabbit, HRP, secondary antibody (polyclonal, #31464, ThermoFisher Scientific, Waltham, MA, USA) at a ratio of 1:5000 in 2% non-fat milk for 2 hours at 4°C. Amersham ECL western blotting detection system (RPN2108, Cytivia) was utilized to visualize protein bands on a myECL Imager (Thermo Scientific). The membranes were then stripped by incubating in a weak stripping buffer (0.1% SDS, 1.5% glycine, 1% polysorbate 20, pH 2.2) twice, for 10 mins each, at room temperature. After another 3 washes in PBST, membranes were incubated in anti-human/mouse/rat β-Actin monoclonal antibody (~45 kDa, clone 937215, #MAB8929-SP, R&D Systems Inc., Minneapolis, MN, USA) at a ratio of 1:500 in 2% non-fat milk, washed 3 times using PBST, then incubated in IgG (H+L) Rabbit anti-Mouse, HRP, secondary antibody (polyclonal, #31450, ThermoFisher Scientific, Waltham, MA, USA) at a ratio of 1:5000 in 2% non-fat milk for 2 hours at 4°C. The membranes were then re-imaged for detection of the β-actin loading control bands. Western blots were performed 3 times, on 3 separately isolated protein samples, to ensure repeatability.

### Irradiation of cells

Cells were plated in 6-well plates at counts of 3.5 x 10^5^ cells/well for SAOS-2, and 2.5 x 10^5^ cells/well for LM6, MG63, and U2OS. After resting overnight, RNA was extracted from no-irradiation (NIR) control cells and remaining cells were treated with radiation as described below. Complete media was changed every 2–3 days.

Radiation treatments were performed under atmospheric oxygen using 6MV X-rays from a Varian 21EX clinical linear accelerator. 5 Gy were administered to cell cultures at a dose rate of ~0.8 Gy/min. A 1 cm bolus was placed over the cells to target treatment to the bottom of the wells.

RNA and protein were extracted on Day 0 (NIR cells), Day 1, Day 3, and Day 6 after radiation and RT-qPCR was used to determine changes in gene expression. Protein concentration was determined using methods previously described. 30ug protein was then combined with 1x Laemmli sample buffer, incubated at 95°C for 5 mins, and loaded onto a NuPAGE 4–12% Bis-Tris mini protein precast polyacrylamide gel (NP0335BOX, Invitrogen, Carlsbad, CA, USA). PageRuler Plus Prestained Protein Ladder (26620, ThermoFisher Scientific, Waltham, MA, USA) was added to one well. 1x MOPS SDS running buffer (NP0001, Invitrogen, Carlsbad, CA, USA) was used for electrophoresis under the following conditions: 50 V for 15 mins, 90 V for 20 mins, and 150 V until complete. The transfer process was performed using methods previously described. The membrane was cut just above the 55 kDa marker, and the top portion was stained to detect phospho-STAT1 (pSTAT1, ~84 & 91 kDa, 1:500, clone 58D6, #9167S, Cell Signaling Technology, Danvers, MA, USA) while the bottom portion was stained to detect the loading control GAPDH (~37 kDa, 1:2000, clone GA1R, #MA515738, Invitrogen, Carlsbad, CA, USA), so membrane stripping was not required.

### Gene knockdown by siRNA transfection

siRNA transfection was performed using GenMute siRNA transfection reagent (SL100568, SignaGen Laboratories, Frederick, MD, USA) following manufacturer’s instructions. Previously published STING siRNA sequences were utilized (S1 Table). MG63 cells were plated in 6-well plates at a count of 5 x 10^5^ cells/well. After resting overnight, cells were treated using either scramble control (SCR) siRNA or STING knockdown siRNA (MG63-SCR and MG63 STING-KD respectively). Forty-eight hrs post transfection, MG63-SCR, and MG63-STING KD cells were exposed to 5 Gy radiation, or no irradiation (NIR). SAOS cells were included as a negative control. RNA was extracted on Day 0 (NIR cells), Day 1, Day 3, and Day 6 after radiation and RT-qPCR was used to determine changes in gene expression.

### Statistics

Concentrations of CCL5 and CXCL10 as determined by ELISA were log transformed prior to comparison between control and cGAMP-treated cells using multiple paired t-tests. Samples measuring below the lower limit of detection (LLD) were set to a value of 0.5*LLD. RT-qPCR data were analyzed by log_2_ transforming fold change (2^-ΔΔCt^). A one-way ANOVA and Dunnett’s or Tukey’s multiple comparisons test were used to compare expression between cell lines, and timepoints post RT to NIR controls. Multiple t-tests were used to compared cGAMP-treated cells to control cells. A two-way ANOVA and Dunnett’s multiple comparisons test was used compare gene expression post radiation between control and STING-KD cell lines. Statistical analysis was performed using Prism 9 for MacOS, v9.4.0 (GraphPad Software). P values less than 0.05 were considered statistically significant. P values for all comparisons made are listed in S2 Table.

## Results

### STING is variably downregulated in OSA cell lines

**Fig 1:**
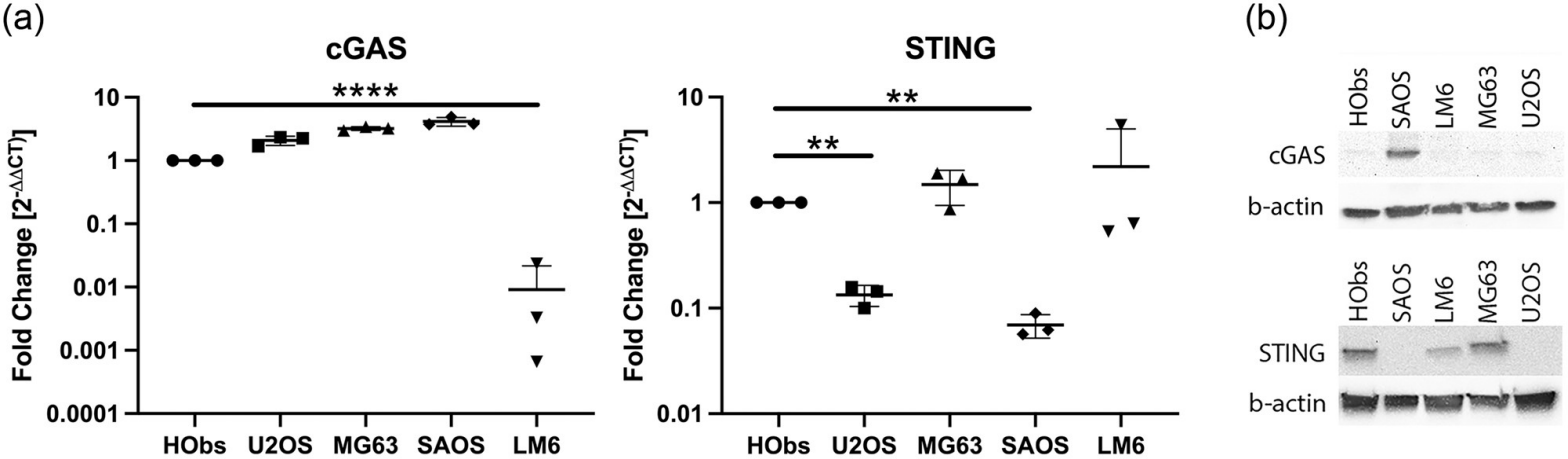
Expression of cGAS and STING by hOSA cell lines relative to hObs. A) cGAS and STING mRNA expression depicted as fold change (2^-ΔΔCt^) in hOSA cell lines relative to hObs. Log_2_(fold change) was compared between hOSA cell lines and hOBs, and comparisons yielding p values < 0.05 are shown. RT-qPCR was performed in duplicate. N = 3. The y-axes are represented on a log_10_ scale. B) cGAS and STING protein expression in hObs and hOSA cell lines was determined Western blot. A representative image is shown, depicting cGAS expression (~62 kDa), STING expression (~35 kDa) and the loading control, β-actin (~45 kDa). Individual data points are plotted, mean and SD are shown. Three independent replicates were performed. ** p < 0.01; **** p < 0.0001.

OSA cell lines including U2OS, MG63, SAOS, and LM6 were evaluated for their baseline expression of cGAS and STING mRNA transcripts relative to hObs. All cell lines except LM6 expressed cGAS in similar or greater amounts compared to hObs (Fig 1a). cGAS expression by LM6 cells was consistently markedly decreased compared to hObs (p < 0.0001). STING expression was consistently decreased in U2OS (p = 0.009) and SAOS (0.001) cell lines, whereas MG63 and LM6 cell lines expressed STING at similar levels compared to hObs (Fig 1a). On Western blot, SAOS cells expressed greater amounts of cGAS protein compared to all other cell lines (Fig 1b). Consistent with RT-qPCR findings, no cGAS protein was detected in the LM6 cell line, and U2OS and SAOS cell lines showed decreased STING protein compared to the other cell lines (Fig 1b).

**Fig 2:**
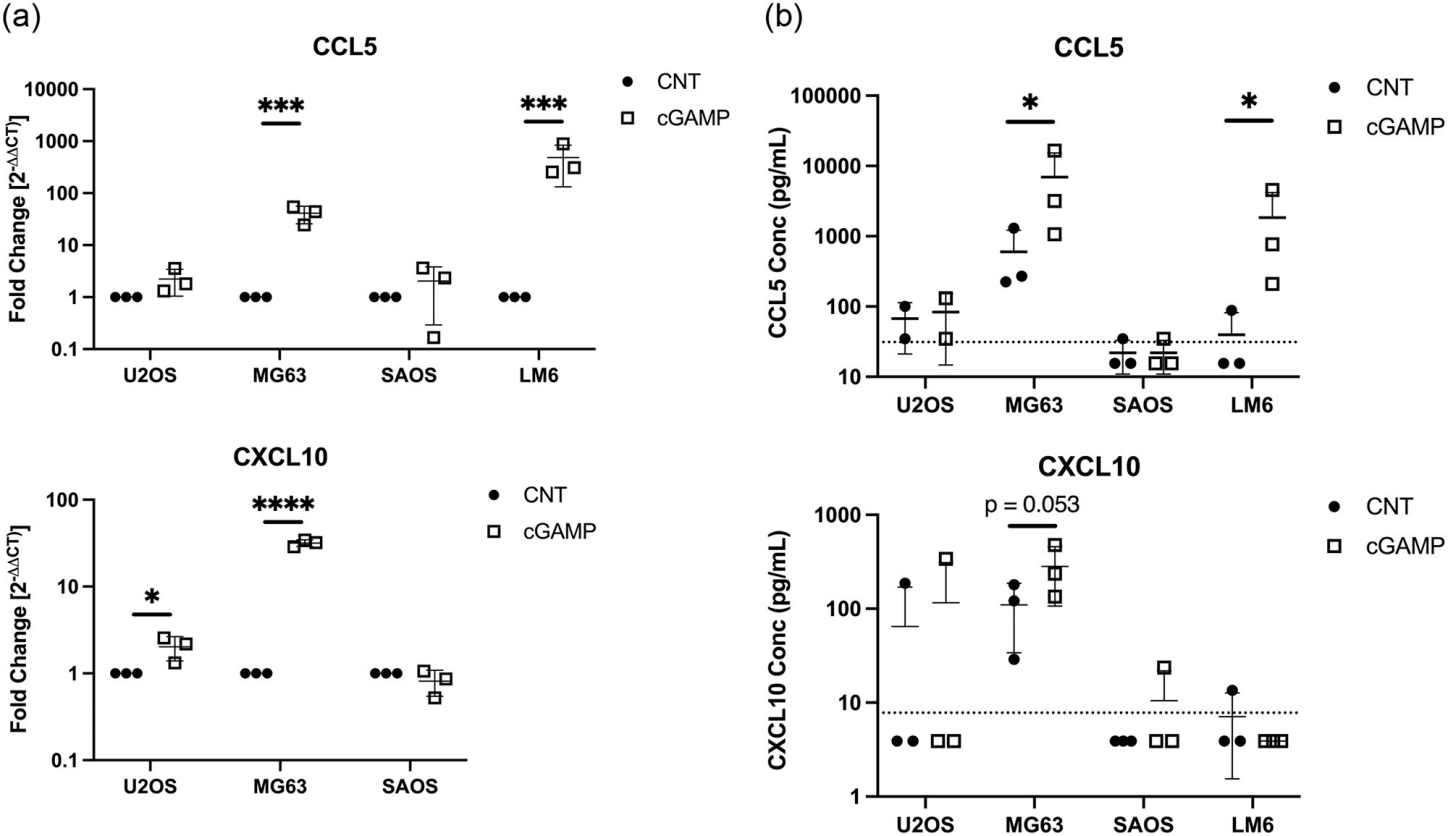
Expression of chemokines induced by STING agonism in hOSA cells. A) CCL5 and CXCL10 mRNA expression depicted as fold change (2^-ΔΔCt^) in cells treated with 2’3’-cGAMP (10 μg/mL for 16 hrs) relative to control cells. RT-qPCR was performed in duplicate. N = 3. B) CCL5 and CXCL10 protein expression in cGAMP-stimulated cell lines (10 μg/mL for 24 hrs) relative to control cells was determined by ELISA. The dashed horizontal line represents the lower limit of detection for the CCL5 (31.2 pg/mL) and CXCL10 (7.8 pg/mL) ELISAs used. ELISAs were performed in duplicate. N = 2–3. Individual data points are plotted, mean and SD are shown. The y-axes are represented on a log_10_ scale. * p < 0.05; *** p < 0.001; **** p < 0.0001.

To confirm that the observed decreased STING expression in U2OS and SAOS cell lines could result in a functional blockade of chemokine expression via this pathway, we incubated cells with the STING agonist 2’3’-cGAMP (10 μg/mL) for 16 hrs prior to RNA isolation, and 24 hrs prior to supernatant collection. As expected, cGAMP exposure did not induce CCL5 expression in STING-deficient cell lines, U2OS and SAOS, relative to control cells (Fig 2a). STING-expressing cell lines such as MG63 (p < 0.001), and LM6 (p < 0.001), showed consistent marked induction of CCL5 with cGAMP exposure. CXCL10 was also increased in cGAMP-stimulated MG63 (p < 0.001) cell lines (Fig 2a). CXCL10 transcripts were absent in both control and stimulated LM6 cells. STING-deficient U2OS cells demonstrated a statistically significant but relatively small increase in CXCL10 expression of only approximately 2-fold in cGAMP-treated cells (p = 0.028), while SAOS cells remained unchanged in their CXCL10 expression.

Similar effects of cGAMP exposure were observed for CCL5 and CXCL10 protein expression amongst the OSA cell lines evaluated (Fig 2b). cGAMP-induced upregulation of CCL5 protein was only observed in the STING-expressing cell lines MG63 (p = 0.020) and LM6 (p = 0.020), and increased CXCL10 protein was only detected in the MG63 cell line, although the increase from control did not reach statistical significance (p = 0.053). CXCL10 protein was primarily undetectable in LM6 cell lines, which mirrored RT-qPCR results. cGAMP did not induce CCL5/CXCL10 protein expression by STING-deficient cell lines U2OS and SAOS.

These findings confirm that STING is downregulated in the U2OS and SAOS cell lines relative to hObs, while MG63 and LM6 cell lines retain expression of STING.

### STING downregulation decreases radiation-induced inflammatory signals produced by OSA cells

Given the critical role of STING in linking DNA damage to innate inflammation and recruitment of immune effector cells, we next sought to determine how STING expression could impact radiation-induced inflammatory mediator expression in OSA cells.

**Fig 3:**
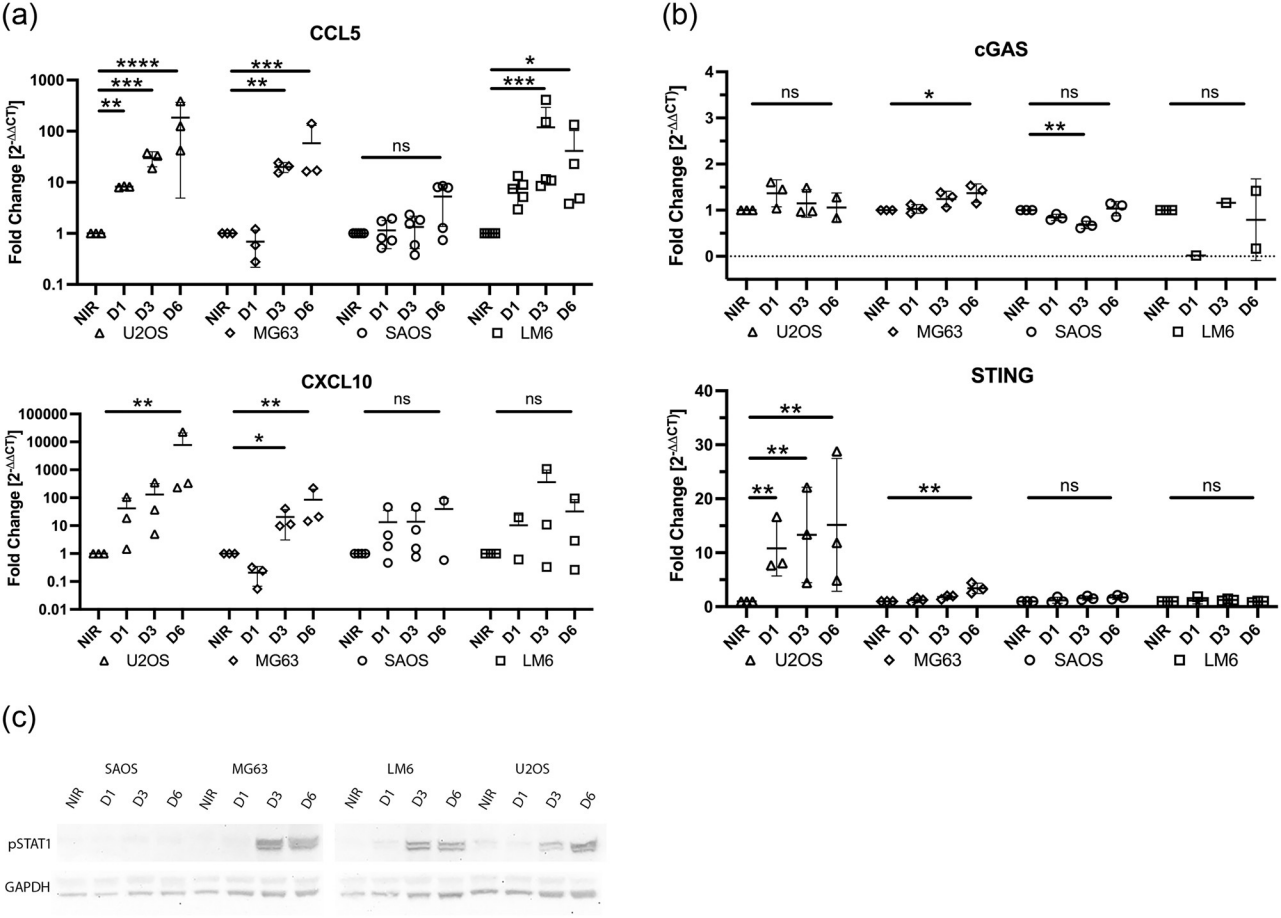
Expression of chemokines induced by 5Gy ionizing radiation in hOSA cells. A) CCL5 and CXCL10 mRNA expression depicted as fold change (2^-ΔΔCt^) at 1 day (D1), 3 days (D3), and 6 days (D6) post radiation exposure, compared to non-irradiated cells (NIR). The y-axis is represented on a log_10_ scale. RT-qPCR was performed in duplicate. N = 3–5. B) cGAS and STING mRNA expression depicted as fold change (2^-ΔΔCt^) at various timepoints post radiation exposure, compared to NIR. RT-qPCR was performed in duplicate. N = 3. C) Phosphorylated STAT (pSTAT) protein expression in hOSA cell lines at various timepoints following radiation treatment compared to NIR control cells performed by Western blot. A representative image is shown, depicting pSTAT expression (~85–90 kDa) and the loading control, GAPDH (~35kDa). Three independent replicates were performed. Individual data points are plotted, mean and SD are shown. ns = not statistically significant; * p < 0.05; ** p < 0.01; *** p < 0.001; **** p < 0.0001.

We exposed OSA cells to 5 Gy ionizing radiation and monitored the expression of CCL5 and CXCL10 at day 1, day 3, and day 6, relative to NIR cells (Fig 3a). Robust increases in CCL5 transcripts occurred from day 1 in U2OS cells, and day 3 in MG63 and LM6 cells. Meanwhile, SAOS cells showed only minor upregulation of CCL5 expression at day 6 post-irradiation. Only MG63 and U2OS cell lines displayed statistically significant upregulation of CXCL10 transcripts, which occurred from day 3 for MG63, and day 6 for U2OS.

Given the unexpected, marked upregulation of CCL5 and CXCL10 by the STING-deficient cell line U2OS, we hypothesized that STING may be induced by radiation exposure in this cell line. We found that while cGAS expression changed minimally and inconsistently in all cell lines after radiation exposure, STING was rapidly and markedly upregulated in U2OS cells by at least 10-fold from day 1 post-radiation onwards (Fig 3b). A small increase in STING expression in MG63 cells was also detected by day 6, while no STING upregulation was detected in either SAOS or LM6 cell lines.

To confirm radiation-induced upregulation of inflammatory signals at the protein level, we measured pSTAT1 expression in protein lysates from OSA cells post-radiation (Fig 3c) [14]. Consistent with RT-qPCR findings from irradiated OSA cells, we observed repeatable increases in pSTAT1 expression from day 1–3 post-radiation in U2OS, MG63, and LM6, cell lines, while expression in SAOS cell lines was absent at all time points.

**Fig 4:**
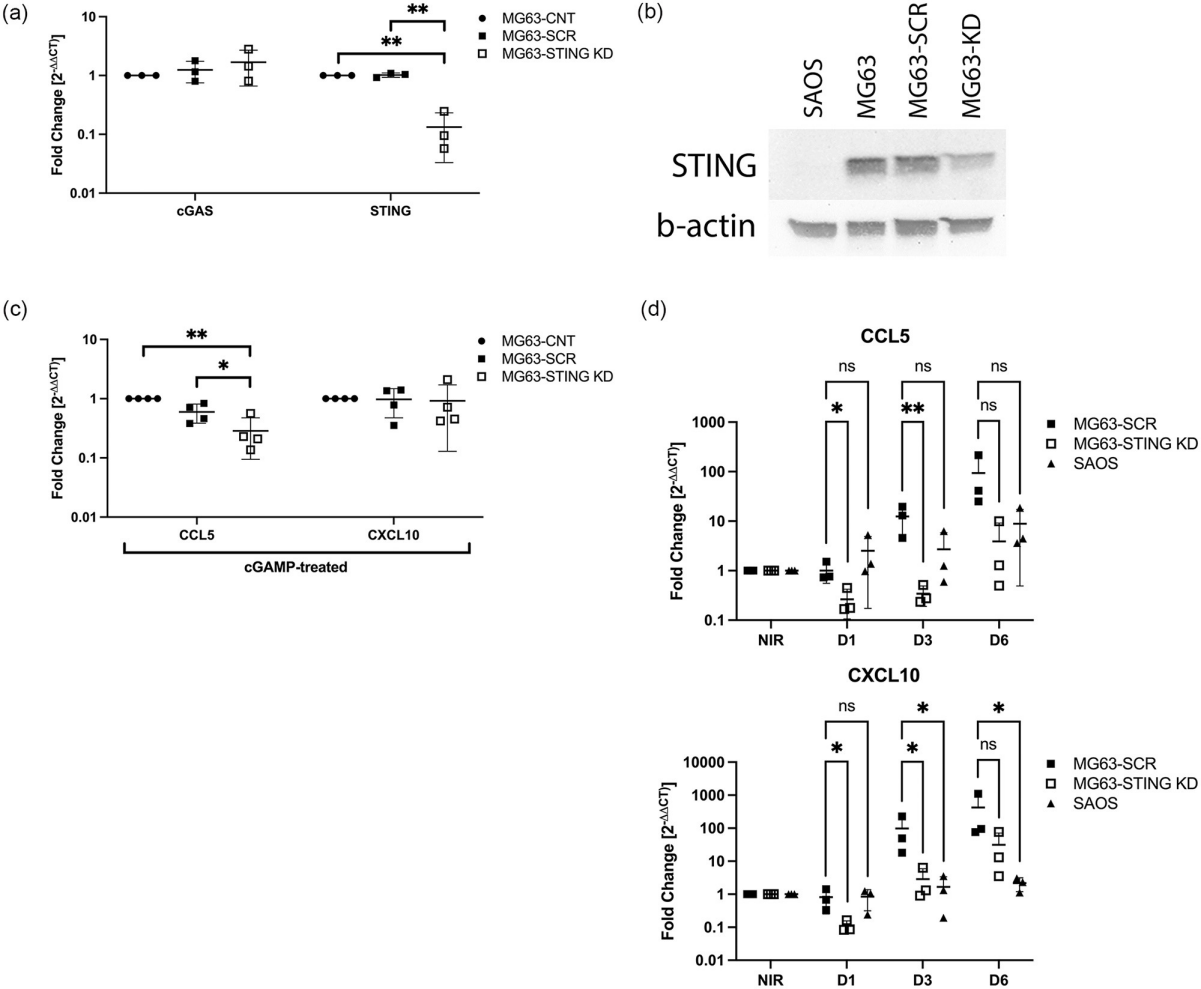
Dependence on STING expression for radiation-induced chemokine expression. A) mRNA expression of cGAS and STING depicted as fold change (2^-ΔΔCt^) in MG63-STING KD cells relative to MG63-SCR and MG63-CNT cells. N = 3. B) STING protein expression in SAOS, MG63-CNT, MG63-SCR, and MG63-STING KD cells by Western blot. A representative image is shown, depicting STING expression (~35 kDa) and the loading control, β-actin (~45 kDa). Three independent replicates were performed. C) CCL5 and CXCL10 expression in MG63-STING KD, MG63-SCR and MG63-CNT cells exposed to 2’3’-cGAMP (10 μg/mL for 16 hrs). N = 4. D) Expression of chemokines by STING-deficient MG63-STING KD and SAOS cells, and STING-expressing MG63-SCR cells after exposure to 5 Gy ionizing radiation. Expression of CCL5 and CXCL10 is shown at 1 day (D1), 3 days (D3), and 6 days (D6) post radiation exposure relative to non-irradiated cells (NIR). Expression is compared between cell lines for each timepoint. RT-qPCR was performed in duplicate. The y-axes are represented on a log_10_ scale. Individual data points are plotted, mean and SD are shown. ns = not statistically significant; * p < 0.05, ** p < 0.01.

To verify that STING downregulation significantly effects inflammatory signals produced by OSA cells after radiation exposure, we used RNA interference to knockdown STING in the STING-expressing MG63 cell line. Previously published siRNAs were confirmed to partially knockdown STING transcript and protein expression in MG63 cells (MG63-STING-KD) relative to scramble-transfected cells (MG63-SCR) and control cells (MG63-CNT; Fig 4a and 4b) [16]. Exposure to 2’3’-cGAMP also revealed decreased CCL5 (but not CXCL10) expression by MG63-STING-KD cells compared to MG63-SCR or MG63-CNT cells (Fig 4c).

We then compared chemokine expression between STING-expressing MG63-SCR cells, STING-deficient SAOS cells, and MG63-STING-KD cells, post-irradiation (Fig 4d). Consistent with prior findings, SAOS cells displayed only minimal increases in chemokine expression at the delayed timepoint of 6 days post-radiation. In contrast, MG63-SCR cells displayed marked increases in CCL5/CXCL10 from day 3 onwards. MG63-STING-KD cells however, demonstrated consistently depressed chemokine expression with levels only increasing above NIR cells at day 6 for CCL5, and day 3 for CXCL10. MG63-STING-KD cells expressed statistically significantly lower levels of CCL5 and CXCL10 at the D1 and D3 timepoints relative to MG63-SCR control cells (Fig 4d).

**Fig 5:**
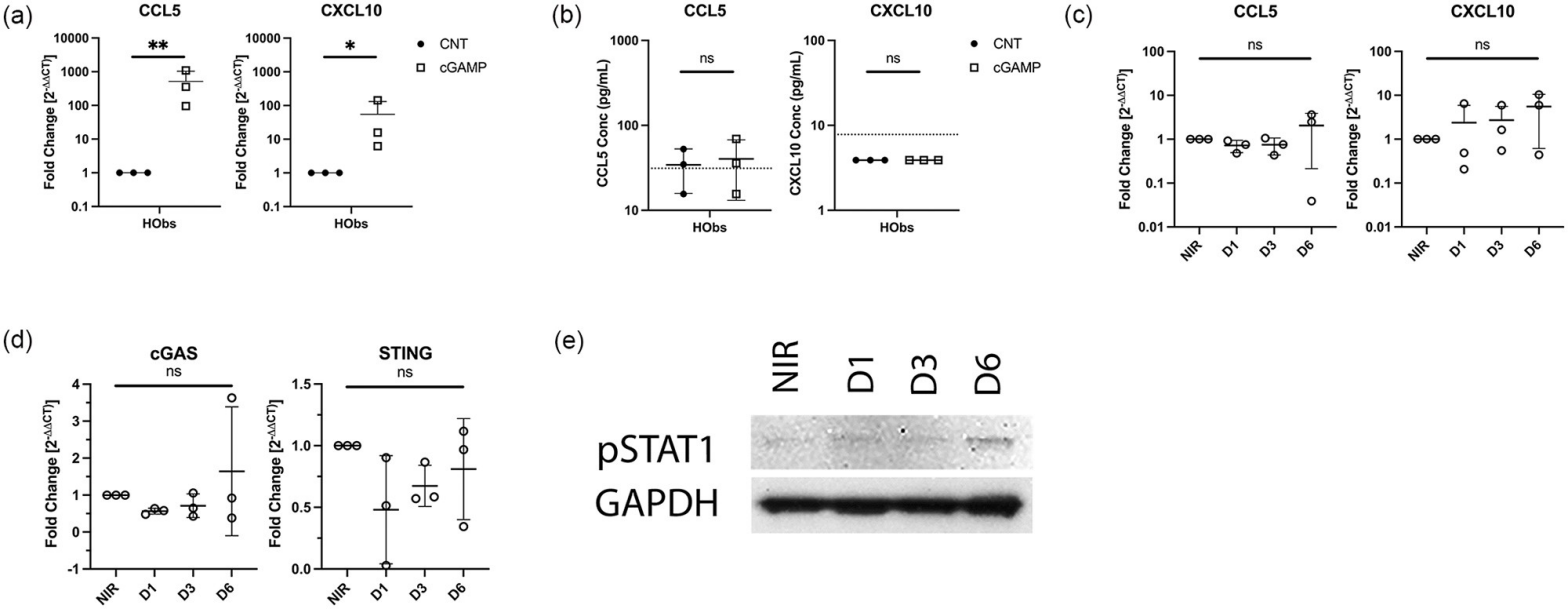
Expression of chemokines induced by STING agonism or ionizing radiation in hObs cells. A) CCL5 and CXCL10 mRNA expression depicted as fold change (2^-ΔΔCt^) in cells treated with 2’3’-cGAMP (10 μg/mL for 16 hrs) relative to control cells. N = 3. B) CCL5 and CXCL10 protein expression in cGAMP-stimulated cell lines (10 μg/mL for 24 hrs) relative to control cells was determined by ELISA. The dashed horizontal line represents the lower limit of detection for the CCL5 (31.2 pg/mL) and CXCL10 (7.8 pg/mL) ELISAs used. N = 3. C) CCL5 and CXCL10 mRNA expression depicted as fold change (2^-ΔΔCt^) at 1 day (D1), 3 days (D3), and 6 days (D6) post radiation exposure, compared to non-irradiated cells (NIR). N = 3. D) cGAS and STING mRNA expression at various timepoints post radiation exposure, compared to NIR. N = 3. RT-qPCR was performed in duplicate. Individual data points are plotted, mean and SD are shown. The y-axes are represented on a log_10_ scale. * p < 0.05; ** p < 0.01. E) Phosphorylated STAT (pSTAT) protein expression in hObs cells at various timepoints following irradiation compared to NIR control cells performed by Western blot. A representative image is shown, depicting pSTAT expression (~85–90 kDa) and the loading control, GAPDH (~35kDa). Three independent replicates were performed.

Finally, we interrogated the hObs cell line to determine the link between STING expression and chemokine production in untransformed cells exposed to radiation. cGAS and STING are expressed in similar amounts to the MG63 cell line and demonstrate a robust increase in CCL5 and CXCL10 transcript expression upon cGAMP stimulation (Figs 1a, 1b and 5a). However, an increase in CCL5 and CXCL10 protein expression could not be detected in cGAMP stimulated hObs cells via ELISA (Fig 5b). Furthermore, despite ample expression of cGAS and STING, we did not identify any increases in CCL5 or CXCL10 in hObs cells exposed to radiation and nor did we identify any modulation of cGAS or STING post radiation (Fig 5c and 5d). These findings suggest additional mechanisms that vary between hOSA cells and hObs cells, may play a role in mediating these chemokine responses.

## Discussion

Findings reported herein reveal a reliance on STING expression for maximum production of CCL5 and CXCL10 by OSA cells after radiation exposure. We observed STING expression to be downregulated in two of the four OSA cell lines, relative to hObs. After radiation exposure, STING-expressing OSA cell lines showed robust increases in downstream chemokine expression while the STING-deficient cell line, SAOS, demonstrated only a subtle increase at Day 6. Despite U2OS cells expressing low levels of STING at baseline, marked increases in STING expression after radiation exposure correlated with a robust increase in CCL5 and CXCL10 expression. Depressed expression of these chemokines in STING-KD MG63 cells after radiation exposure further supports the role of STING in mediating the link between radiation-induced DNA damage and CCL5/CXCL10 expression. Given the importance of CCL5 and CXCL10 in mediating an anti-tumor immune response [17–20], these findings raise additional hypotheses regarding the significance of OSA cell intrinsic STING expression to effect radiation immunomodulation *in vivo*.

Intratumoral cGAS-STING signaling appears to be an important pathway for promoting anti-cancer immunity [10, 21–24]. cGAS detects the presence of dsDNA within the cytoplasm and is therefore critical for sensing intracellular virus and bacterial particles, as well as cytoplasmic dsDNA fragments that arise in states of genomic instability and DNA damage. Subsequent cGAMP formation and STING activation result in type I IFN production and trigger upregulation of innate and adaptive immune mediators [6, 10, 11]. Consequently, cGAS-STING signaling places neoplastic cells at risk of initiating anticancer immunity by cGAS sensing of cytoplasmic dsDNA fragments in cancer cells exhibiting genomic instability [25]. The induction of CCL5 and CXCL10 chemokine expression induced by STING signaling appears particularly critical for recruiting immune effector cells such as CD8+ T lymphocytes and NK cells into the tumor microenvironment [4, 5]. Therefore, downregulation of STING by cancer cells is thought to be an immunoevasion strategy [26–29]. Genotoxic agents such as ionizing radiation can also cause DNA fragmentation and consequential detection of cytosolic dsDNA by cGAS, which triggers downstream STING signaling [14]. Intratumoral STING expression appears critical for the ability of localized radiation therapy to induce systemic anti-tumor immunity (abscopal effect) [13, 30, 31]. While there is some evidence that cGAMP derived from tumor cells can diffuse into neighboring host immune cells, thus overcoming tumor cell STING deficiency in some models, cancer cell intrinsic cGAS expression remains important for cGAMP formation [4, 6, 12, 25]. Given the role of STING in mediating cellular immunity to intracellular DNA viruses, several studies have correlated STING downregulation in cancer cells with increased cytotoxicity from DNA oncolytic viruses [26, 32]. Therefore, in addition to the relevance of STING downregulation to immune responses triggered by genomic instability and radiation-induced DNA damage, data herein may also predict susceptibility to DNA oncolytic viruses.

Herein, we found STING expression to be necessary for maximal radiation-induced production of CCL5 and CXCL10 by OSA cells *in vitro*. MG63 and LM6 OSA cell lines both expressed STING at equivalent levels to that of hObs, and consequently displayed robust increases in downstream CCL5 expression after cGAMP treatment and radiation exposure. Increased CXCL10 was also detected in treated MG63 cells, however LM6 cells exhibited very low or absent CXCL10 in both control and treated cells. The reason for this discrepancy between CCL5 and CXCL10 in the LM6 cell line remains unclear. The duration of OSA cell exposure to cGAMP of 16hrs prior to mRNA isolation was in line with similar previous publications utilizing timepoints between 9–24 hrs [15, 18]. However, earlier timepoints may help to characterize immediate effects of cGAMP on RNA expression. Interestingly, despite cGAMP stimulation inducing similar CCL5 transcript upregulation in cells expressing higher levels of STING (hObs, MG63 and LM6), and CXCL10 transcript upregulation in hObs and MG63 cell lines, increases in CCL5 and CXCL10 protein were only evident in neoplastic cells. In contrast, hObs cells increased transcript expression of CCL5 and CXCL10 in response to cGAMP stimulation, without concurrent increases in protein expression. This difference in mRNA/protein correlation between neoplastic and non-neoplastic cells could be due to variability in protein half-life, the regulation of protein translation, or the detection of different protein variants [33]. Additionally, while we focused on CCL5 and CXCL10 expression as a metric of STING signaling, similar to previous studies, IFNβ is another important mediator of STING activity that was not evaluated in this study [4, 12–14].

Despite U2OS cells expressing very little STING at baseline, they were capable of upregulating STING after radiation exposure, which correlated with upregulation of CCL5/CXCL10 expression. LM6 cells displayed robust increases in chemokine expression after radiation exposure despite having extremely low cGAS expression. These findings indicate additional mediators of DNA damage detection such as ATM and IFI16 may contribute to STING signaling in these cell lines [34]. Furthermore, additional STING-independent mechanisms of DNA sensing via DNA-PK activity, may also be at play [35]. We subsequently performed STING knockdown and demonstrated inhibition of CCL5 expression in cGAMP-stimulated and irradiated MG63 cells. While CXCL10 expression in cGAMP stimulated MG63-STING KD cells did not differ significantly from that of the MG63-CNT and -SCR control cell lines, we did observe a significant inhibition of CXCL10 expression at day 1 and day 3 post irradiation in STING-KD cells compared to -SCR controls. This apparent discrepancy may be due to a suboptimal timepoint for quantifying CXCL10 transcripts post STING agonism with cGAMP. Taken together, these data suggest that STING expression at baseline, or rapid upregulation of STING, is necessary for maximal production of CCL5 and CXCL10 post-irradiation.

Our findings show variability in STING expression amongst OSA cell lines; from equivalent to that of hObs to significantly downregulated. Previous studies have also found STING to be frequently downregulated in cancer cells derived from ovarian carcinoma [36], colorectal cancer [26], and melanoma [27], amongst others. Loss of STING expression may also correlate with clinical variables and genotype. For example, STING downregulation in colorectal carcinoma cells was more frequent in tumors of stage II or higher [26]. Interestingly, LKB1 loss in KRAS-mutant non-small cell lung cancers (NSCLC) was also associated with epigenetic silencing of STING expression, which promoted cell survival by mediating tolerance of cytoplasmic DNA accumulation [37]. The mechanism of STING downregulation in OSA cells was not evaluated in this study; however, prior reports describe epigenetic silencing, increased STING degradation, cGAMP hydrolysis, altered post-translational modifications, and inhibition of STING signalosome assembly [29, 36, 37]. Our data also revealed variable cGAS transcript and protein expression in our hOSA cell lines, with upregulation in the SAOS cell line, and down regulation in LM6. This is a particularly interesting finding since the LM6 cell line is derived from several rounds of selecting for metastatic SAOS cells from lung lesions. Interestingly, reports of cGAS expression in other tumor types are variable with both up- and down-regulation demonstrated in colon cancer, melanoma, and ovarian cancer [26, 27, 38, 39]. Schadt et al. specifically highlighted the relevance of tumor cell intrinsic cGAS expression, and correlated expression levels with CD8+ T lymphocyte infiltration into microsatellite-stable colorectal cancers [25]. Therefore, our findings of frequent STING downregulation and cGAS upregulation in OSA cell lines are in line with studies in other tumor types.

Finally, several microenvironmental factors that have not been modelled in this study are certain to further modulate radiation-induced immune responses. Much of the intratumoral STING signaling may occur in tumor infiltrating myeloid cells such as macrophages and dendritic cells, in certain tumors. As previously discussed, the presence of host myeloid cells may negate the need for tumor cell intrinsic STING expression [4, 6, 12, 25]. However, the proportion of infiltrating host myeloid cells varies between tumors, necessitating an understanding of the consequences of STING signaling in tumors cells themselves. Additional microenvironmental variables such as the presence or absence of immunomodulatory cells (e.g. MDSCs) and inhibitory ligands (e.g. PD-L1), and factors correlating to tumor immunogenicity (e.g. tumor neoantigen burden) may also affect radiation-induced immunity to OSA [40–42]. Consequently, our findings are indicative of one piece of a large and complex puzzle that requires further study.

These data reveal a dependence on STING signaling in OSA cells for radiation-induced expression of CCL5 and CXCL10. Such findings necessitate the future study of correlations between OSA cell STING expression and radiation-induced immune responses.

## Supporting information

S1 Raw images Annotated, uncropped and unadjusted Western blot images. Image processing performed on raw Western blot images to create the final Figs 1b, 3c, 4b, and 5e.
(PDF)

S1 Table Primer and siRNA sequences. (DOCX)

S2 Table P values for all comparisons made. (XLSX)
