# Supplementary material for: Effect of stimulator of interferon genes (STING) signaling on radiation-induced chemokine expression in human osteosarcoma cells
Source: PLoS One. 2023 Apr 20;18(4):e0284645. doi: 10.1371/journal.pone.0284645 (PMC10118169; doi:10.1371/journal.pone.0284645)
Supplement: S1 Raw images — Image processing performed on raw Western blot images to create the final Figs 1b, 3c, 4b, and 5e. (PDF) [file pone.0284645.s001.pdf]

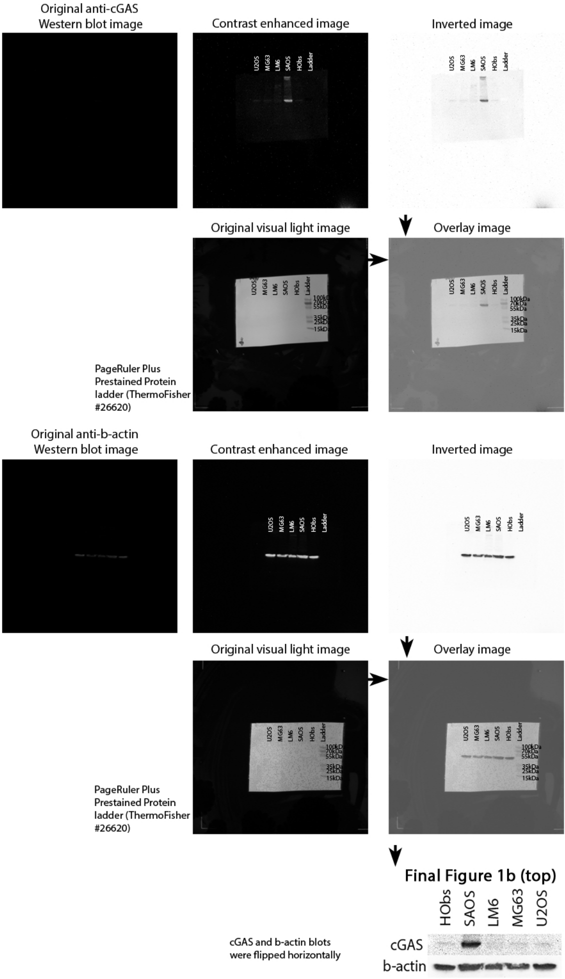

Original anti-cGAS Western blot image
Contrast enhanced image
U2OS MG63 LM6 SAOS HObs Ladder
Inverted image
U2OS MG63 LM6 SAOS HObs Ladder
Original visual light image
U2OS MG63 LM6 SAOS HObs Ladder
Overlay image
U2OS MG63 LM6 SAOS HObs Ladder
100kDa 70kDa 55kDa 35kDa 25kDa 15kDa
PageRuler Plus Prestained Protein ladder (ThermoFisher #26620)
Original anti-b-actin Western blot image
Contrast enhanced image
U2OS MG63 LM6 SAOS HObs Ladder
Inverted image
U2OS MG63 LM6 SAOS HObs Ladder
Original visual light image
U2OS MG63 LM6 SAOS HObs Ladder
Overlay image
U2OS MG63 LM6 SAOS HObs Ladder
100kDa 70kDa 55kDa 35kDa 25kDa 15kDa
PageRuler Plus Prestained Protein ladder (ThermoFisher #26620)
Final Figure 1b (top)
HObs SAOS LM6 MG63 U2OS
cGAS
b-actin
cGAS and b-actin blots were flipped horizontally

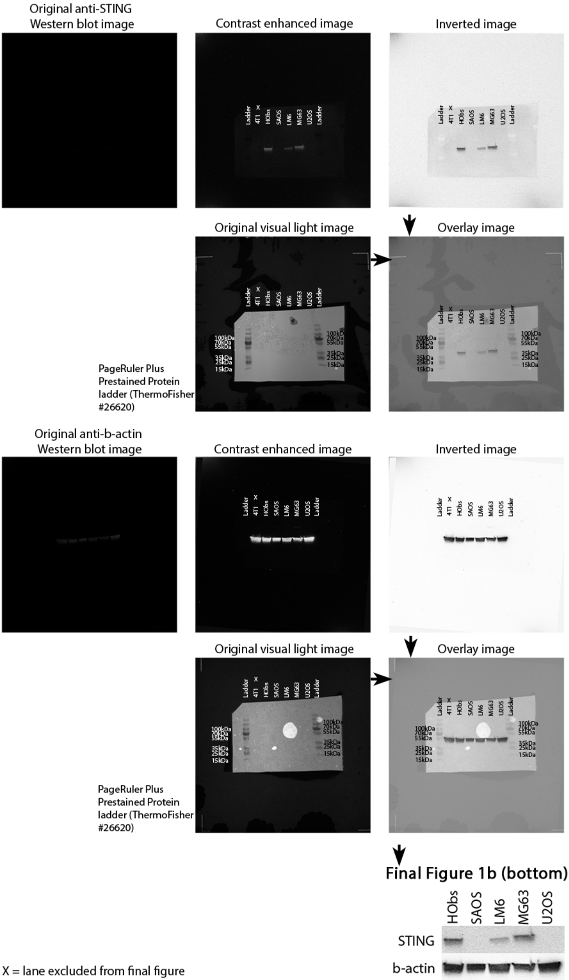

Original anti-STING Western blot image
Contrast enhanced image
Inverted image
Ladder 4T1 x HObs SAOS LM6 MG63 U2OS Ladder
Original visual light image
Overlay image
100kDa 70kDa 55kDa 35kDa 25kDa 15kDa
PageRuler Plus Prestained Protein ladder (ThermoFisher #26620)
Original anti-b-actin Western blot image
Contrast enhanced image
Inverted image
Original visual light image
Overlay image
PageRuler Plus Prestained Protein ladder (ThermoFisher #26620)
Final Figure 1b (bottom)
HObs SAOS LM6 MG63 U2OS
STING
b-actin
X = lane excluded from final figure

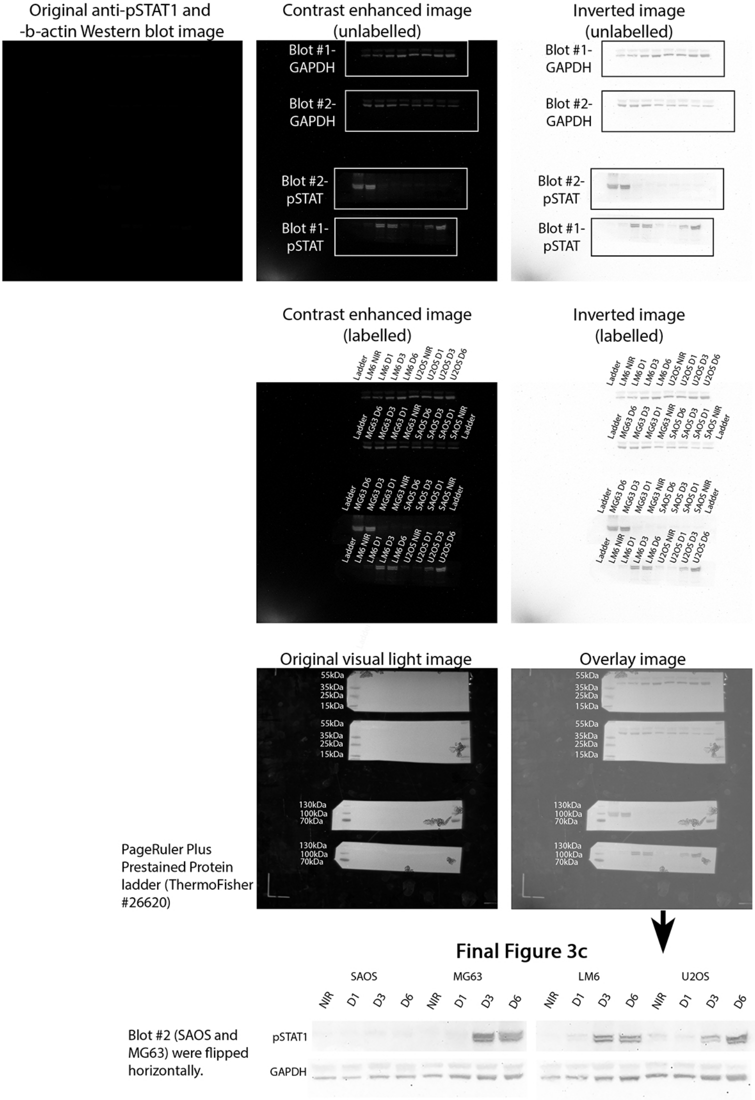

Original anti-pSTAT1 and -b-actin Western blot image
Contrast enhanced image (unlabelled)
Inverted image (unlabelled)
Blot #1- GAPDH
Blot #2- GAPDH
Blot #2- pSTAT
Blot #1- pSTAT
Contrast enhanced image (labelled)
Inverted image (labelled)
Ladder LM6 NIR LM6 D1 LM6 D3 LM6 D6 U2OS NIR U2OS D1 U2OS D3 U2OS D6
Ladder MG63 D6 MG63 D3 MG63 D1 MG63 NIR SAOS D6 SAOS D3 SAOS D1 SAOS NIR Ladder
Original visual light image
Overlay image
55kDa
35kDa
25kDa
15kDa
130kDa
100kDa
70kDa
PageRuler Plus Prestained Protein ladder (ThermoFisher #26620)
Final Figure 3c
SAOS
MG63
LM6
U2OS
NIR D1 D3 D6 NIR D1 D3 D6 NIR D1 D3 D6 NIR D1 D3 D6
pSTAT1
GAPDH
Blot #2 (SAOS and MG63) were flipped horizontally.

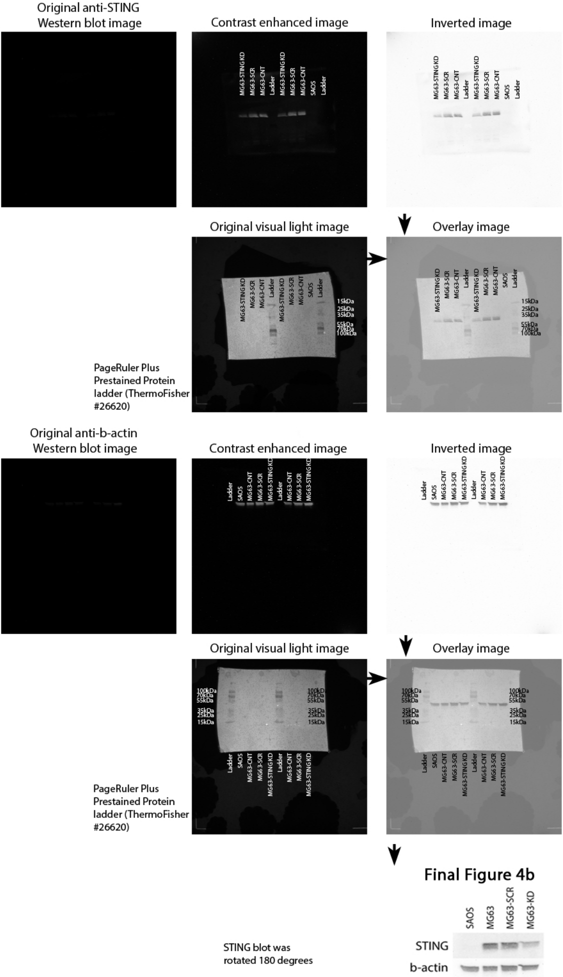

Original anti-STING Western blot image
Contrast enhanced image
MG63-STING KD
MG63-SCR
MG63-CNT
Ladder
MG63-STING KD
MG63-SCR
MG63-CNT
SAOS
Ladder
Inverted image
Original visual light image
Overlay image
15kDa
25kDa
35kDa
55kDa
70kDa
100kDa
PageRuler Plus Prestained Protein ladder (ThermoFisher #26620)
Original anti-b-actin Western blot image
Contrast enhanced image
Ladder
SAOS
MG63-CNT
MG63-SCR
MG63-STING KD
Ladder
MG63-CNT
MG63-SCR
MG63-STING KD
Inverted image
Original visual light image
Overlay image
100kDa
70kDa
55kDa
35kDa
25kDa
15kDa
PageRuler Plus Prestained Protein ladder (ThermoFisher #26620)
Final Figure 4b
SAOS
MG63
MG63-SCR
MG63-KD
STING
b-actin
STING blot was rotated 180 degrees

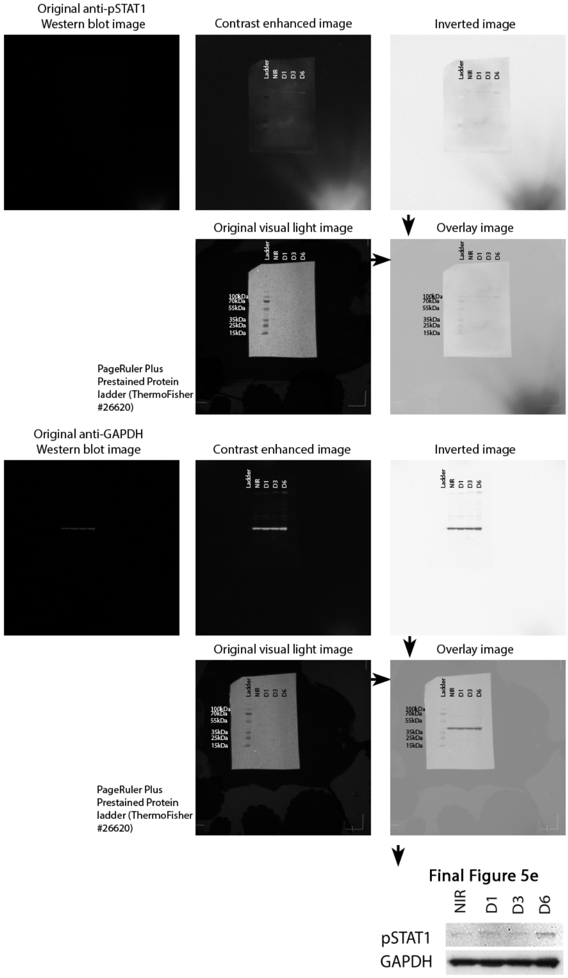
Original anti-pSTAT1
Western blot image
Contrast enhanced image
Inverted image
Ladder NIR D1 D3 D6
Original visual light image
Overlay image
100kDa
70kDa
55kDa
35kDa
25kDa
15kDa
PageRuler Plus
Prestained Protein
ladder (ThermoFisher
#26620)
Original anti-GAPDH
Western blot image
Contrast enhanced image
Inverted image
Original visual light image
Overlay image
PageRuler Plus
Prestained Protein
ladder (ThermoFisher
#26620)
Final Figure 5e
NIR D1 D3 D6
pSTAT1
GAPDH
