# Supplementary material for: Effect of stimulator of interferon genes (STING) signaling on radiation-induced chemokine expression in human osteosarcoma cells
Source: PLoS One. 2023 Apr 20;18(4):e0284645. doi: 10.1371/journal.pone.0284645 (PMC10118169; doi:10.1371/journal.pone.0284645)
Supplement: S1 Table — (DOCX) [file pone.0284645.s002.docx]

**S2 Table- Primer and siRNA sequences**

| **Primer sequences** | | | | |  |
| --- | --- | --- | --- | --- | --- |
| **Gene** | **Forward primer (5’- 3’)** | **Reverse primer (3’- 5’)** | **RefSeq#** | **Product size (bp)** | |
| TBP | TTGGGTTTTCCAGCTAAGTTCT | CCAGGAAATAACTCTGGCTCA | NM_001172085.1 | 140[1] | |
| cGAS | TGGTGAAAGGGGTTGTGGAC | GTGCAGAAATCTTCACGTGCT | NM_138441.3 | 114 | |
| TMEM173 (STING) | GAGCAGGCCAAACTCTTCTG | TGCCCACAGTAACCTCTTCC | NM_198282.4 | 178[2] | |
| CCL5 | CCAGCAGTCGTCTTTGTCAC | CTCTGGGTTGGCACACACTT | NM_002985.2 | 54[3] | |
| CXCL10 | GTGGCATTCAAGGAGTACCTC | TGATGGCCTTCGATTCTGGATT | NM_001565.4 | 198[4] | |
| **siRNA sequences** | | |  |  |  |
| **Gene** | **Forward RNA oligonucleotide (5’- 3’)** | **Reverse RNA oligonucleotide (3’- 5’)** |  |  |  |
| Scramble | rArGrUrArUrArUrGrArCrCrGrCrUrArGrArUrCrUrU | rGrArUrCrUrArGrCrGrGrUrCrArUrArUrArCrUrUrU |  |  |  |
| TMEM173 (STING) | rCrUrGrGrCrArUrGrGrUrCrArUrArUrUrArCrArUrU | rUrGrUrArArUrArUrGrArCrCrArUrGrCrCrArGrUrU |  |  |  |

1. Lemma S, Avnet S, Salerno M, Chano T, Baldini N. Identification and Validation of Housekeeping Genes for Gene Expression Analysis of Cancer Stem Cells. PLoS One. 2016;11(2):e0149481. Epub 2016/02/20. doi: 10.1371/journal.pone.0149481. PubMed PMID: 26894994; PubMed Central PMCID: PMCPMC4760967.

2. Ma Z, Jacobs SR, West JA, Stopford C, Zhang Z, Davis Z, et al. Modulation of the cGAS-STING DNA sensing pathway by gammaherpesviruses. Proc Natl Acad Sci U S A. 2015;112(31):E4306-15. Epub 2015/07/23. doi: 10.1073/pnas.1503831112. PubMed PMID: 26199418; PubMed Central PMCID: PMCPMC4534226.

3. Harding SM, Benci JL, Irianto J, Discher DE, Minn AJ, Greenberg RA. Mitotic progression following DNA damage enables pattern recognition within micronuclei. Nature. 2017;548(7668):466-70. Epub 2017/08/02. doi: 10.1038/nature23470. PubMed PMID: 28759889; PubMed Central PMCID: PMCPMC5857357.

4. Kitajima S, Ivanova E, Guo S, Yoshida R, Campisi M, Sundararaman SK, et al. Suppression of STING Associated with LKB1 Loss in KRAS-Driven Lung Cancer. Cancer Discov. 2019;9(1):34-45. Epub 2018/10/10. doi: 10.1158/2159-8290.CD-18-0689. PubMed PMID: 30297358; PubMed Central PMCID: PMCPMC6328329.
